# Implementation of Pharmacogenomics and Artificial Intelligence Tools for Chronic Disease Management in Primary Care Setting

**DOI:** 10.3390/jpm11060443

**Published:** 2021-05-21

**Authors:** Patrick Silva, David Jacobs, John Kriak, Asim Abu-Baker, George Udeani, Gabriel Neal, Kenneth Ramos

**Affiliations:** 1 Texas A&M Health Science Center, 8441 Riverside Pkwy, Bryan, TX 77807, USA; ricksilva@tamu.edu (P.S.); abu-baker@tamu.edu (A.A.-B.); udeani@tamu.edu (G.U.); gneal@tamu.edu (G.N.); 2 Goldblatt Systems, LLC., 5151 E. Broadway Blvd., Tucson, AZ 85711, USA; jkriak@molecdx.com

**Keywords:** pharmacogenomics, polypharmacy, chronic disease, medication management, electronic medical record, artificial intelligence

## Abstract

Chronic disease management often requires use of multiple drug regimens that lead to polypharmacy challenges and suboptimal utilization of healthcare services. While the rising costs and healthcare utilization associated with polypharmacy and drug interactions have been well documented, effective tools to address these challenges remain elusive. Emerging evidence that proactive medication management, combined with pharmacogenomic testing, can lead to improved health outcomes and reduced cost burdens may help to address such gaps. In this report, we describe informatic and bioanalytic methodologies that integrate weak signals in symptoms and chief complaints with pharmacogenomic analysis of ~90 single nucleotide polymorphic variants, CYP2D6 copy number, and clinical pharmacokinetic profiles to monitor drug–gene pairs and drug–drug interactions for medications with significant pharmacogenomic profiles. The utility of the approach was validated in a virtual patient case showing detection of significant drug–gene and drug–drug interactions of clinical significance. This effort is being used to establish proof-of-concept for the creation of a regional database to track clinical outcomes in patients enrolled in a bioanalytically-informed medication management program. Our integrated informatic and bioanalytic platform can provide facile clinical decision support to inform and augment medication management in the primary care setting.

## 1. Introduction

The management of chronic diseases in the primary care setting often involves polypharmacy challenges that often drive considerable healthcare utilization and costs. While the term polypharmacy is used inconsistently in the literature [1], for the purposes of this report, we are making reference to clinical instances where five or more medications are used concurrently. Analysis of the Observational Health Data Sciences and Informatics data set has documented that 10% of diabetes, 24% of hypertension and 11% of depression patients followed a treatment pathway that was unique among 250 million cases [2], thus yielding a daunting number of permutations in drug combinations. This increasing armamentarium necessitates individualized care plans, a challenging task for primary care practitioners managing complex patient populations. OptumRx has estimated that polypharmacy affects about 15% of the US population and costs over $175 B per year [3]. As such, polypharmacy is believed to have increased healthcare cost burdens in recent years by ~30% [4]. Reduced adherence to drug therapy regimens and heightened incidence of adverse drug reactions (ADRs) represent major challenges for polypharmacy patients, including at-risk patients with multiple comorbid conditions.

An estimated 15 million people 65 years of age and older face a polypharmacy challenge, with nearly 50% of them using at least one unnecessary medication [5]. The prevalence of potential hepatic cytochrome (CYP) enzyme-mediated drug–drug interactions has been estimated to be as high as 80% [6], with elder adults considered to be more susceptible to problematic drug interactions due to declining levels of hepatic and renal functions [7]. In a recent report, 56% of a study population prescribed (es)citalopram showed underlying drug–drug and drug–gene interactions, which would be difficult for a practitioner to address absent of pharmacogenomic testing [8]. Further complicating the optimization of complex pharmacotherapies, individual variations in response to the same medication can fluctuate over three orders of magnitude [9]. These issues collectively contribute to over 2 million documented ADRs in the US and over 100,000 deaths [10]. Analysis of the genetic variation in response to drugs and ADRs has been enabled somewhat by the Pharmacogenomics Knowledge Base (PharmGKB), Pharmacogenomics Research Network (PGRN), and the Clinical Pharmacogenetics Implementation Consortium (CPIC) [11,12,13]. However, data linkages and documentation of the full diversity of clinical phenotypes associated with rare or emergent variants remain a significant hurdle. One of the largest pharmacogenomic targeted exome sequencing studies conducted to date [14] has shown that 96.2% of patients in a cohort of 5424 had CPIC Level A actionable variants, with half of the variants identified in the population identified as novel variants [15]. In a different but concordant report by Van Driest et al., 98% of the study population carried CPIC actionable variants [16].

ADRs are major drivers of healthcare utilization, and precision interventions designed to address these deficits provide tremendous opportunities for improved health outcomes and reduced costs. These challenges are especially prevalent among rural and socioeconomically disadvantaged populations, with most studies to date largely limited to retrospective observations and data mining. These approaches pose data linkage limitations that preclude longitudinal assessment of the natural history of polypharmacy and chronic disease progression at the individual level [17]. As such, robust studies of polypharmacy and the contribution of genetics to drug response have continued to be sparse.

Pharmacogenomics (PGx) is a discipline that focuses on a genome-wide assessment of how individual genes alone or in combination with other loci affect individual responses to drug treatment. PGx combines pharmacology (the science that focuses on the uses, effects, and modes of action of drugs) and genomics (the study of structure, function, evolution, and mapping of genomes) to develop effective, safe medication regimens tailored to an individual’s genetic makeup [13]. PGx can aid in the prediction and stratification of who may benefit from a medication, who may not respond at all, and who may experience adverse reactions. Clinical use and reimbursement of pharmacogenomic testing remains challenging, largely due to a dearth of evidence supporting clinical utility and cost effectiveness. While clinical decision support is recognized [18] as a key component for the successful implementation of pharmacogenomic testing, widespread utilization of pharmacogenomic testing is not commonplace [19]. Pharmacogenomic-based studies can inform how single nucleotide polymorphisms (SNPs) and other variations in the human genome correlate with disease, drug response, and the occurrence of clinically significant phenotypes [20]. SNPs, the most common type of genetic variation found in humans and the most commonly tested variant affecting drug response [20], may be present between genes or within genes and their regulatory sequences. While most SNPs do not affect health, some may be linked to disease or help to predict an individual’s response to a particular drug [20]. As such, SNP-based pharmacogenomic analysis can highlight specific targets and their impact on the subject’s medication blood levels and response. Genomic and pharmacogenomic data combined with personal health and psychosocial data may be effectively used to support providers with treatment-related decisions in the management of chronic disease patients.

Here we present proof-of-concept for a clinical care protocol with the potential to predict and confirm a subject’s response to their medication based on chief complaints and symptom functionality, specific medication-associated genomic data on receptors and transporters, and measurement of drug levels. Evidence is presented that a computational rendering of a patient’s complaints and medications alone can be useful in the identification of symptoms with pharmacologic root-cause and that genotypes and pharmacokinetic information can be used computationally in a way that is practical for guiding prescribing choices in a primary care setting.

## 2. Materials and Methods

### 2.1. Clinical Environment and Process

A Texas A&M Interprofessional Pharmacogenomics (IPGx) Clinic is being established as part of the Texas A&M Family Medicine Program, a clinical practice serving diverse and underserved populations with chronic disease burden, including a high prevalence of polypharmacy.

**Figure 1:**
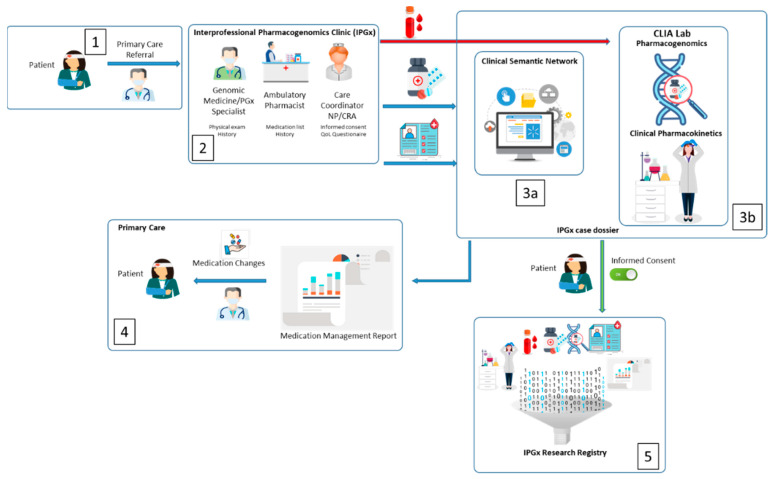
Interprofessional Pharmacogenomics (IPGx) Model. **1**. Referral of polypharmacy patient to the IPGx clinic. **2**. Interprofessional team collects relevant medical history with an emphasis on information related to chief complaints, which also includes a transition of care history from primary care to the IPGx. This information is analyzed using the Clinical Semantic Network to identify complaints of possible pharmacological root cause. **3a**. When warranted, pharmacogenomic profiling is performed. **3b**. When warranted, pharmacokinetic profiling is performed. **4**. A medication management report citing complaints of potential pharmacological root causes and suggested alternative medications or adjustments to drug regimen is provided to the referring physician. **5**. If patient chooses to give informed consent, all clinical data, bioanalytic data and biological specimens are entered into a pharmacogenomic research registry (clinical-genomic database).

A digital continuity of care document/file with the contents listed in Table 1 above is produced by the primary care electronic medical record for digital importation into the (Clinical Semantic Network) CSN (Figure 1, step 2). This represents the baseline information required for completion of the data analysis underlying the IPGx care model (Figure 1, step 3a). Basic medical and family history information is collected followed by a physical exam and collection of blood and/or buccal swabs for processing by a CAP-CLIA bioanalytic laboratory (Figure 1, step 3b). A medication management report citing complaints of potential pharmacological root causes and suggested alternative medications is provided to the referring physician (Figure 1, step 4). Patients are offered an opportunity to participate in a research registry underlying the IPGx database (Figure 1, step 5), and if opting into the registry, administered baseline validated quality of life questionnaires in digital format.

### 2.2. Medical Record Analysis and the Clinical Semantic Network

The first piece of the artificial intelligence phase in the IPGx model is an analysis of symptoms residing in electronic medical records that might be indicative of suboptimal or problematic medication regimens. The Clinical Semantic Network (Goldblatt Systems, Inc., Tucson, AZ, USA [21] is a computable medical record that enables facile analysis of symptoms and complaints imported from the Texas A&M Primary Care medical record (eClinical Works), in an HL7 clinical document architecture (CDA, see Table 1 IPGx continuity of care (CoC) data). The IPGx CoC data in the CDA Clinical Document Architecture [22] includes chief complaints such as drug side effects and symptoms, known diagnoses, and medications prescribed. This information is exported from the electronic health record into the CSN to render case specific data computable by the CSN [21].

The CSN is built on a commercial grade software that is tiered from Oracle, DOM, Hibernate, and Java. It maps relational data to a domain model. The data structure is computationally tractable and configured to enable the application of AI in terms of predictive analytics.

### 2.3. Bioanalytic Phase

The bioanalytic phase of the IPGx model involves interrogation of specific and actionable pharmacogenomic targets (per CPIC guidelines) and confirmation of genotype impact on the subject’s steady state blood levels of medication.

#### 2.3.1. Clinical Pharmacogenomics

SNPs identified with genomic and pharmacogenomic analyses combined with personal health and psychosocial data may be used to develop a model for prediction of disease outcome as well as an aid to physicians with clinical management [23]. The Molecular Dx pharmacogenomic (PGx) assay targets an extensive list of medications and therapeutic symptoms. For low daily volume and fast turn-around time, the MolecularDx Comprehensive PGx panel is utilized (Table 2). Primers designed to amplify specific genetic variations (SNPs, insertions, deletions, multi-nucleotide polymorphisms) in genes coding for pharmacogenes or their regulatory elements are listed in Table 2.

This technology uses TaqMan Genotyping Assays [24] to target 90 PGx-related SNPs plus CYP2D6 copy number. Genotyping is performed on the Applied Biosystems QuantStudio 12K Flex. For higher daily volume and lax turn-around time, a custom-designed SARS-CoV-2 research diversity array can be utilized. Whole blood with no centrifugation is extracted using QIAamp DNA Blood Mini Kit (Cat. # 51106) used on QiaCube. TaqMan Genotyper v1.6 software was used to make genotype calls. Calls are manually reviewed by two pharmacists with pharmacogenomic expertise and agreed upon before reporting.

#### 2.3.2. Clinical Pharmacokinetics

Candidate pharmacologic symptoms coupled with genotypes that portend drug–drug and drug–gene interactions can be further validated by the measurement of steady state blood concentrations of the medications of interest. Under the IPGx protocol, target drugs corresponding to the drug–gene pairs in Table 2 and their metabolites are measured utilizing a validated liquid chromatography mass spectrometry assay. Such results were not entered into the virtual exercise presented in this report, but are available for use in clinical practice.

### 2.4. Synthesis and Reporting

The CSN deconstructs and enhances a medication identification procedure utilizing the medication’s molecular weight, excretion pathways, ATC class, volume of distribution, bioavailability, elimination half-life, anticholinergic burden, steady state, and CYP_450_, or transporter pathways. In this light, the CSN enabled the development of a polypharmacy report that can be utilized by a clinician at point of care to get a holistic, yet cogent snapshot of symptoms, complaints, diagnoses, and medications that might reflect drug–drug and drug–gene interactions. This function generates a medication management summary report that identifies high probability and actionability per CPIC guidelines of drug–gene and drug–drug interactions at the root cause for select symptoms. These relationships can ideally be confirmed by genotyping and/or clinical pharmacokinetic assays.

## 3. Results

### 3.1. Clinical Environment and Process

Polypharmacy patients and patients demonstrating symptoms and complaints that might be indicative of possible medication interactions are referred to the IPGx clinic for evaluation by the attending clinician (Figure 1, step 1). Patients are not required to consent to the registry to receive the bioanalytic workup and medication management care; registry participation is optional and not a condition of care. The program entails a process of stepwise progression of electronic medical record analysis toward pharmacokinetic ground truth to inform primary care practitioners. The first step consists of a clinically aware computational analysis such that entry of complaints into the patient’s record, updates the rendering of complaints that match the known side effects (from First Data Bank) of drugs taken by the patient. The second step strengthens these associations if a pharmacokinetic model of the medications renders potential instances of pathway overload (Epocrates). Next, the CSN can further strengthen these associations by identification of pharmacogene variants of known clinical significance that are consistent with the list of candidate side-effects or pathway overload. Finally, pharmacokinetic data is incorporated to distinguish among and validate instances of drug–gene or drug–drug interactions.

### 3.2. Medical Record Analysis and the Clinical Semantic Network

The first step is a computational and semantic comparison of case history to the medications the patient is taking. This is powered by DrugBank [25], Epocrates [26], and Lexicomp [27] to tally the subset of symptoms that are present and known to occur as side effects of the medications the patient is taking. At a clinical informatics level, cough with fever might be connected/mapped to curated ontologies such as SNOMED (Systematized Nomenclature of Medicine [28]) about SARS-CoV-2, or pneumonia in a weighted fashion, by subject matter experts who then have the capability to markedly enhance this highly navigable information. A net result is that a SNOMED identification can be established with multiple attributes built into the CSN system. Other analogies in the CSN include instances when a chief complaint is entered such that the system knows which history of present illness questions should be used to interrogate. The technology works much in the same manner as Google can predict shopping preferences based on user actions.

For the purposes of this communication, we created a virtual patient with medication, pharmacogenomic, and side effect profiles that were aggregated drawing from previous experiences with a number of real-life clinical cases. The clinical characteristics assessed included:

- Number of side effects/complaints/diagnoses identified in the patient and believed to be related to his/her current medication regimen,
- Number of medications possibly contributing to the identified/diagnosed side effects,
- Number of drug metabolic pathways identified as being potentially overloaded,
- Number of drug metabolic pathways identified as borderline overloaded,
- Number of medications with pharmacogenomic profiles,
- Number of medications putting the patient at risk for serotonin syndrome,
- Number of medications putting the patient at risk for QT prolongation,
- Number of anticholinergic medications.

**Figure 2:**
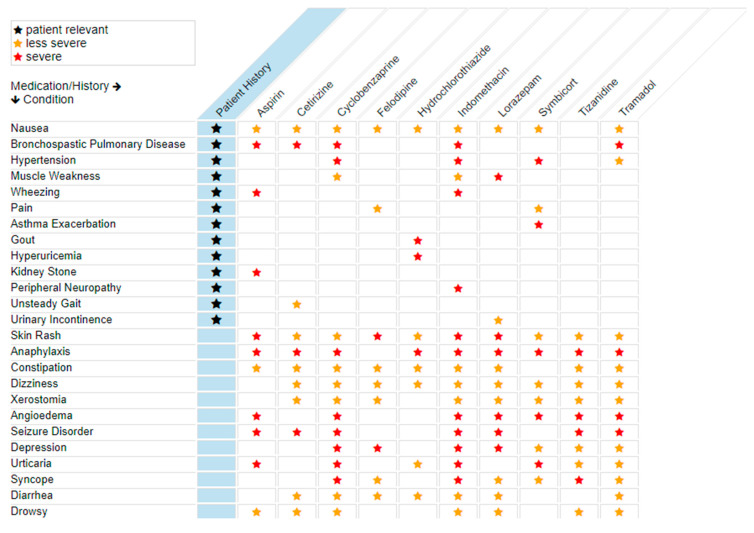
Side Effects Dashboard. List of symptoms indicating potential pharmacological origin.

**Figure 3:**
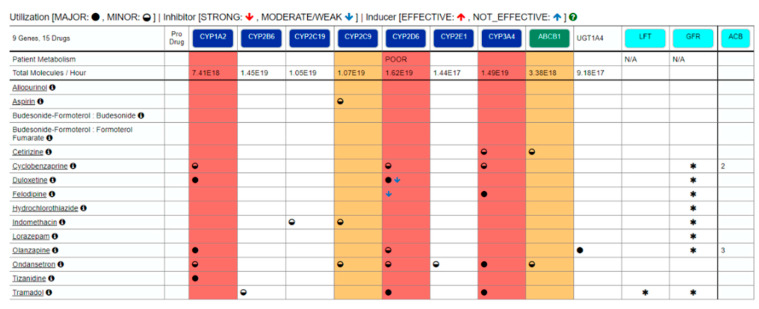

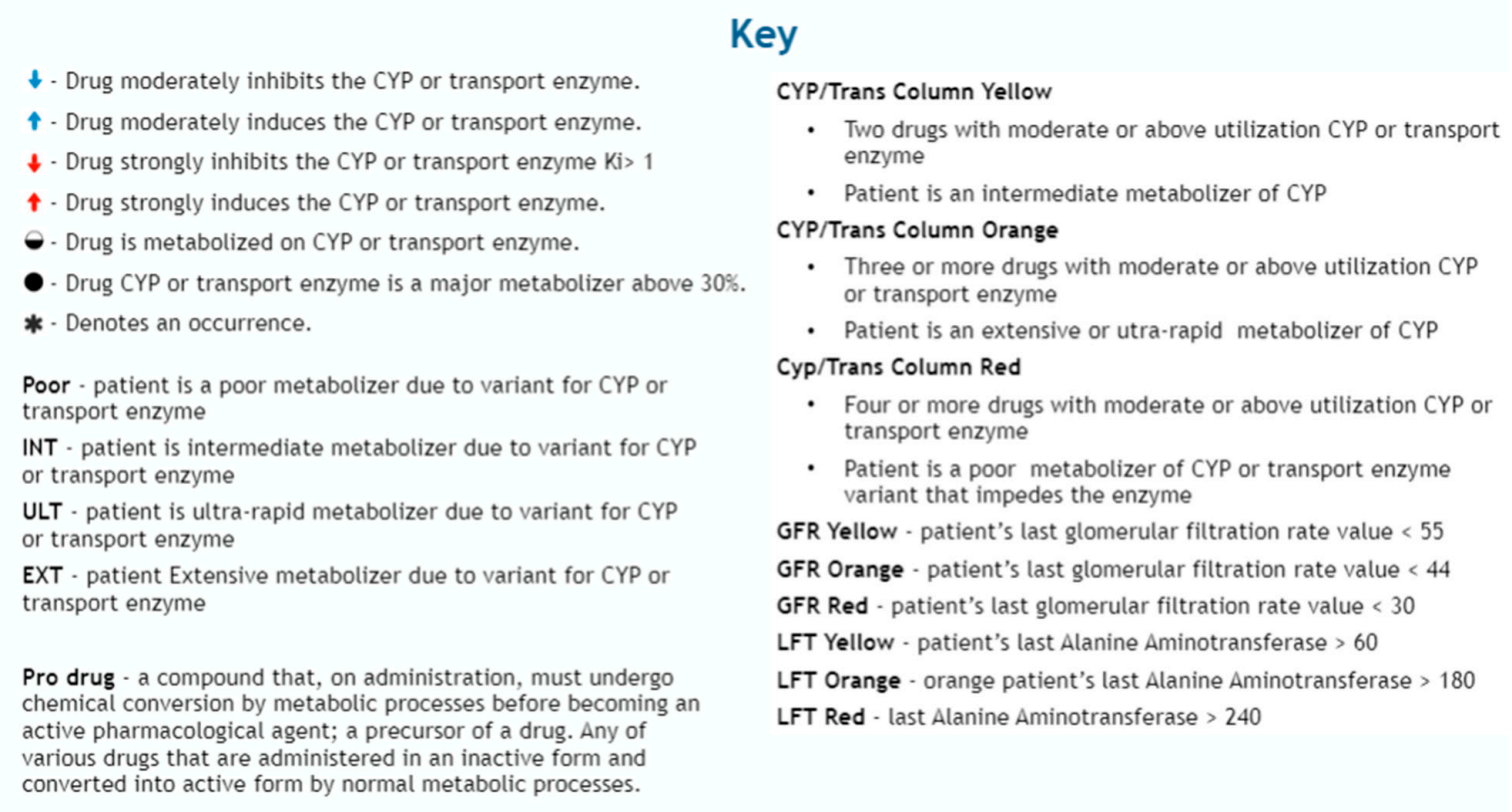
Medication and Pharmacokinetic Pathway Summary. The green rectangles are a few salient transporters, and the light blue box represents anticholinergic burden. The left most vertical column denotes the patient’s medications. Vertically, the column beneath named alleles in red indicates that the respective pathway could be overloaded. Panel B Key.

At this step, a side-effects dashboard is created by the CSN utilizing any drug in the First Databank to distill complaints that could be of pharmacological origin. Figure 2 provides a summary of selected computational clinical findings for the virtual patient which are also a rendering of the complaints from Table 1, that correspond to the subset of complaints that are also known side effects of the medications the patient is taking. Those side effects, as initially rendered prior to pharmacogenomic profiling, may not inherently be of pharmacological origin, or may arise due to drug–drug interactions, or as a result of drug–gene interactions. Pharmacogenomic testing and pharmacokinetic testing can reveal whether these complaints are rooted in drug–gene or drug–drug interactions. The CSN can be contrasted from step-and-fetch functionality of most electronic medical records by the interconnectivity of medical terms. Those medical terms are connected in a neural-like network of semantic associations that effectively represent knowledge and contextual awareness of potentially related data elements in a patient record. The network consists of nodes representing objects and arcs which describe the relationship between those objects. Semantic networks can categorize the objects in various forms and can link those objects making it particularly useful in an electronic health record which can utilize and act on computable data. Interconnecting a patient’s clinical content (phenotypes) with this form of health care knowledge gives the data in these relationships actionable context. There is a pharmacokinetic modeling dimension in this analysis that examines the repertoire of medications a patient has been prescribed and that models these data based on known pathways for those medications to assess drug–drug interactions that might result from pathways that are excessively taxed by virtue of the combination of medications (Figure 3).

This dashboard can incorporate correlative associations (complaints-drug side effects) and bioanalytic associations (PGx genotypes and predicted or measured pharmacokinetic). As such, this computationally rendered dashboard provides useful insight on the potential root cause of complaints both before and after bioanalytic analysis is entered into the CSN case record.

### 3.3. Bioanalytics

Pharmacogenomics is used to assess the impact of individual pharmacogenomic variants on how subjects respond to their medication by evaluating specific medication receptor targets as well as transporter functionality [29]. The dashboard design incorporates Clinical Pharmacogenetics Implementation Consortium (CPIC) guidelines and the knowledge-bases contained in PharmGKB and PharmVar to provide a cogent front-end presentation of case-relevant and actionable pharmacologic considerations for use by the clinician at point-of-care. The report reflects a comprehensive analysis of known pharmacogenomic knowledge through the filter of established consensus medical guidelines. For illustrative purposes, Table 3 presents a list of CYP2D6 haplotypes that the CSN is configured to dynamically incorporate into the rendering of the pharmacogenomic analysis. The CSN is capable of incorporating all variants of known clinical significance. The patient scope varies and is dynamically adjusted to the variants that are presented by the instrumentation. The CYP2D6 haplotype call is made from the core variants for each haplotype and all other variants are verified as constant relative to that haplotype so variants of unknown significance are not presented as normal.

### 3.4. Virtual Patient A

Patient A is a 60-year-old African American female with a ten-year history of depression, schizophrenia, and chronic pain who is referred to the IPGx Clinic by her primary care physician for a polypharmacy consult. She began complaining of worsening shifts in her mood and increasing feelings of worthlessness and sadness for the past six-months. She stated that there have been to changes in her family life and that her work shifts had ended a year before. She lives with her husband of 30 years and has two pets. The patient stated that she does not understand why she feels sad all of the time and unable to enjoy life the way she used to after her depression, schizophrenia, and pain had been so well controlled with medications. She stated that her family commented that she is more “irritable” and “angry all the time”. Upon further questioning, she noted that her physician had been making adjustments to her medications and prescribed cyclobenzaprine for rigidity and duloxetine for the worsening feelings of depression six months ago. She denies having any other medical problems at this time and any known allergies to medications. The patient reports taking duloxetine, tramadol, ondansetron, cyclobenzaprine, and olanzapine as prescribed, and denies using over the counter medications, herbal supplements, or illicit drugs. Her vital signs were all within normal limits and her physical examination was unremarkable. The patient consents to pharmacogenomic and pharmacokinetic testing and opts to join the pharmacogenomics registry. A blood sample is drawn and buccal swabs are collected for analysis. The patient returns to clinic after one month for follow up and discussion of findings. The results of the metabolic panel and blood cell counts were unremarkable. Pharmacogenomic testing is conducted and shows that the patient is a poor CYP2D6 metabolizer. Figure 2 presents screenshot depictions of side-effects—a listing of patient reported symptoms and complaints attributed to potential pharmacological root causes (denoted by black stars) compared to known side effects of the medications the patient has been prescribed (denoted by red and yellow stars, for moderate and severe side effects, respectively).

## 4. Discussion

### 4.1. Integration with Primary Care

The IPGx program has leveraged health care policy mandates calling for health data standardization protocols [30]. The transitioning of the IPGx CoC data set (Table 1) into the CSN is somewhat amenable to HL7 continuity of care data CDA formats. These data can be output in a readily computable format from most medical records, and have proven to be tractable and scalable in the IPGx model. The side effects table (Figure 2) for composite clinic case represented by patient A were compiled from a medical history, in the form of a CDA, composed of data enumerated in Table 1 that were extracted from a primary care electronic medical record (eClinical Works) from which patients are being referred for an ongoing pilot project digitally linking the IPGx with primary care. Future work will examine and confirm the utility of using CoC HL7 CDAs using data from other providers and health systems that refer patients to the IPGx clinic. Most current electronic medical record vendors will have standard capability to produce a CoC CDA similar or identical to the one used here. The return of results and medication management recommendations to primary care are a work-in-progress and currently presented in the form of a PDF report that can be appended to the electronic medical record at the referring primary care clinic. However, the CSN retains “clinical awareness” meaning that the semantic linkage between relevant threads in phenotypes->(complaints->potential side-effects)->genotypes->pharmacokinetic ground truth for a given patient are not lost. One existing constraint is that this functionality and the underlying data structures are unique to the CSN and not readily transferrable to any know electronic medical record beyond standard SNOWMED nomenclatures.

A current challenge in primary care environments with populations of polypharmacy and polydisease burden is managing the increased complexity of medication repertoires and standards of care that do not account for this emergent complexity. Some medical records have alerts for potential drug–drug interactions, but none of these computational tools are linked to what the patient is actually experiencing, and as such, they do little to distinguish among complaints caused by disease versus those potentially caused by medications. The status quo leaves the primary care with a dearth of tools to respond to these noisy considerations in an environment with personalized and precision strategies increasingly necessary to avoid ADRs.

### 4.2. Medical Record Analysis and the Clinical Semantic Network

Nausea and depression are symptoms and complaints that the CSN identified in the side effects table as potentially having a pharmacological root cause (Figure 2). The side effects table provides an emerging view of phenotypes that may have a pharmacologic root cause, based on First Data Bank side effects, and warrant further pharmacogenomic and pharmacokinetic analysis of a patient and their case. The combination of worsening symptoms and new medications in Patient’s A dashboard suggests that these symptoms may result from overburdening of the CYP2D6 pathway, an oxidative drug metabolizing pathway utilized by 25% of medications, and a common nexus for ADRs in polypharmacy [31,32]. This assessment is consistent with the finding that many of the medications taken by Patient A (duloxetine, tramadol, ondansetron, cyclobenzaprine, and olanzapine) interact with the CYP2D6 metabolic pathway. Duloxetine is a recognized inhibitor of CYP2D6, thus potentially increasing circulating drug levels for agents such as tramadol that are metabolized by this pathway. Duloxetine, olanzapine, cyclobenzaprine, and ondansetron are also utilizers of CYP1A2, and may be collectively contributing to overload of this metabolic pathway (refer to Figure 3). Several of these agents also utilize the CYP3A2 and CYP3A4 pathways, thus compounding the level of taxation of several alternative oxidative metabolism pathways. In the top left header bar of Figure 3, the dashboard indicates there are 15 drugs, corresponding to 10 genes/pathways of interest in this patient’s medication regimen and that two alternatives to ondansetron (granisetron, palonosetron) should be considered to offload CYP metabolic pathways and possibly improve the patient’s symptoms and optimize response to drug therapy.

Electronic health record alerts, computerized order entry systems, and pharmaceutical box warnings are insufficient in helping physicians at point of care to identify critical drug–drug or drug–gene interactions that might result from polypharmacy [31]. In this case, CYP1A2, CYP2D6, and CYP3A4 may be overburdened (Figure 3) and perhaps overloaded to a point of significant clinical consequence. In this patient’s case, the inclusion of drug–drug interactions in the assessment is complicated by a pre-existing history of dementia and chronic pain, disorders that can themselves present with symptoms of depression. We recognize that ordering a pharmacogenomic test is currently not within established clinical guidelines at this time. In fact, our research registry was established in order to generate clinically relevant evidence to support expanded use of pharmacogenomic testing in chronic disease management and polypharmacy. Again, the medical record analysis is simply an exercise in computational filtering of the symptoms with potential pharmacologic origins from the broader noise contained within the medical and complaint history—a tool that is likely useful in primary care even without deployment of a downstream bioanalytic program.

### 4.3. Bioanalytics

Patient A was designated as a poor metabolizer variant of CYP2D6 into the CSN to establish the utility of layering genotype information onto the pathway analysis dashboard and to incorporate this information in triangulating symptoms with pharmacological root cause to generate medication alternatives. A recent analysis of CYP2D6 genotypes in an Austrian population evaluated in a family practice setting revealed that the metabolizer status of patients taking medications metabolized by CYP2D6 [32] would be clinically actionable in 16% of cases. A 2016 report by Bush et al. focused on variation in 82 pharmacogenes in a cohort of 5000 clinical subjects, and CYP2D6 was identified among the most polymorphic gene present [15]. In their study, over 96% of subjects had one or more CPIC Level A actionable variant identified and more than a third had three or more actionable variants, suggesting that these variations may influence the clinical care of affected patients over their lifetime. In a similar study, Van Driest et al. [16] reported that the number of CYP2D6 variants is highest among African-Americans [15], as seen in Patient A. Accordingly, implementation of our informatic and bioanalytic platform would place critical information at the fingertips of primary care and ambulatory care pharmacy providers. Under the working premise of our virtual case, genotyping confirmed that Patient A had a CYP2D6* 17 variant of CYP2D6 that made her a poor metabolizer. To gain further insight and to validate the functional significance of the findings, a pharmacokinetic assay of CYP2D6 metabolized medications would be ordered to hyperlink steady state levels in the dashboard under the black circle with the letter “i” next to the drug name in the pathway analysis dashboard (Figure 3). This would be done to establish with certainty if the patient has elevated steady state levels of duloxetine and tramadol that not only overload CYP pathways, but may also be material contributors to the chief complaint profile and polypharmacy burden for the virtual patient.

Patient A is taking five medications that utilize a low functioning variant of CYP2D6: duloxetine, tramadol, ondansetron, cyclobenzaprine, and olanzapine. Virtual Patient A notes depression, which could be attributable to several medications this patient is using, including cyclobenzaprine and tramadol. Cyclobenzaprine, a medication that creates a high anticholinergic burden, can exacerbate depression, and could be contributing to CYP1A2, CYP2D6, and CYP3A4 overload. This medication is at the nexus of many of the issues confronting this patient and warrants consideration for alternative medications or supervised deprescribing. If nausea is persistent in the face of a CYP2D6 poor metabolizer with an already overloaded pathway, the system informs the clinician to consider to replace ondansetron with granisetron which lowers metabolic burden at CYP2D6.

This patient has an anticholinergic burden (ACB) score of 5, which is high [33] and could be contributing to adverse effects. Of note is the fact that ondansetron, tramadol, and duloxetine add serotonergic stress and the potential for QT prolongation in our virtual patient. In this instance, the dashboard was further annotated with predicted phenotypes associated with a known actionable variant of CYP2D6 (CYP2D6* 17) that is a poor metabolizer (Figure 3; pursuant to CPIC guidelines). This would augment a genotype annotated and refined computational rendering of a list of side effects with a likely pharmacological root cause. The decision support would identify tizanidine as a potentially less problematic alternative to cyclobenzaprine, or even deprescribing cyclobenzaprine and monitoring the patient. Further, the medications pathway dashboard identifies ondansetron as another potential contributor to CYP2D6 overload (but not depression directly per se) Another therapeutic option that emerges from this case is reconsidering tramadol. Tramadol is metabolized at CYP2D6. It can interact with duloxetine from a drug–drug interaction perspective. Tramadol can induce nausea. If it is clinically determined that Tramadol is required for pain management then a blood drug level is recommended to optimize dosage. If not, tapering and seeking an alternative is reasonable. In this case, stiff person syndrome is in the differential diagnosis due to the burdened serotonin system. In this case, the CSN identifies and presents complaints (depression) that might have pharmacologic root cause (serotonergic burden) likely due to a CYP2D6 variant rendering the patient a poor metabolizer of ondansetron and cyclobenzaprine. In our virtual case, drug–drug–gene interactions were identified electronically from historical clinical data and confirmed with a bioanalytic workup. Specific recommendations for alternative medications included in the final report may help resolve an otherwise disorderly and noisy interplay of polypharmacy, genetic variation, and history of present illness.

## 5. Conclusions

### 5.1. Challenges and Realities

The most obvious challenge with clinical roll-out of the IPGx model is that most of the bioanalytic methodologies described herein are not currently reimbursed by payers and disappointingly, out of reach for most primary care practices. The triggers for genotyping the patients in our test case were rather compelling cases with symptomatology that could with relative ease be attributed to pharmacological root causes: CYP2D6 overload, ACB, and drug–drug and drug–gene interactions. However, this practice is not currently a reimbursable use case for ordering pharmacogenomic testing or clinical pharmacokinetic assays. As such, the methodology described is currently impractical for implementation across the healthcare system due to reimbursement constraints for nearly all private and Medicare insurance policies. The IPGx registry program provides a strategy to measure the positive impact of medication management to deconvolute chronic disease and polypharmacy burdens in a clinically actionable manner and provide evidence of the value-based approach.

Haga and colleagues have published rich commentary [34,35,36], and some primary research, on clinical outcomes in populations in which pharmacogenomic testing has been implemented. The chicken-and-egg paradox to further outcomes research is the dearth of patients for whom pharmacogenomic testing is ordered because of a limited reimbursement landscape [37], and the data linkage challenges posed by efforts to document the public health impact of medication choices. Inherently, genotyping will likely continue to be viewed as having questionable clinical utility absent the grounding provided by measurements of actual drug levels and the clinical actionability that can be inferred when combining genotyping and patient chief complaints.

The evidence versus usage paradox described is the underlying rationale for creation of our IPGx registry. The goal is to collect evidence that these bioanalytic methods are cost effective and can improve outcomes in cases where chronic disease burden and polypharmacy are detrimental to health. Grant funding is often a key component of expanded pharmacogenomic testing beyond the narrow scope provided by the healthcare system in the US. The Vanderbilt University [18] and Duke University Health Systems [38] have robust, interprofessional clinical pharmacogenomic programs, but it is unclear the degree to which access to unreimbursed bioanalytical technology constrains the scope and scale of their efforts to study pharmacogenomic testing at scale, in a clinical setting.

### 5.2. Significance to ADRs

It has been reported that about 2/3 of ADRs are attributable to drug–drug interactions and about 1/3 to drug–gene interactions [39], so a diagnostic battery would ideally inform both of these endpoints. Indeed, the CSN medical record analysis process enables identification of clinically relevant, and inherently actionable, elevated drug levels and/or clinically relevant pharmacogenomic variants. The final medication management report produced by the CSN can reflect suspected medication effects, and provide grounding for both drug–drug and drug–gene interactions. In complex cases, the final report is refined by IPGx clinical staff through an interprofessional consultation among clinical pharmacists, the attending genomic medicine specialist, and the primary care physician to produce a report listing medication management considerations for the referring physician. Such recommendations might include alternative medications to reduce anticholinergic burden or load on specific cytochrome P450 pathways, or deprescribing. In essence, the IPGx platform distills vast electronic health record, genotype, and pharmacokinetic information into an informed, understandable, and actionable set of medication management considerations.

The workflows and reporting for the IPGx and the interface between IPGx and primary care were thoughtfully constructed. Disruptions of workflows or additional work can be a nonstarter for research in a primary care environment and the same holds true for piloting new care models. The upshot of the IPGx platform is that (1) the medical informatic analysis can reveal potential ADR signals in standard continuity of care information sets using the Clinical Semantic Network at the front end, and (2) can be complemented with the bioanalytical analysis from that patients’ pharmacogenomic and clinical pharmacokinetic workup, at the back end of the IPGx encounter. The IPGx model can serve as a force multiplier for the primary care physician in managing their most complex cases driving healthcare utilization by “de-noising” dense medical histories and complementing the analysis with bioanalytic ground truth, to provide cogent actionable data to inform prescribing choices.

### 5.3. Bioanalytics and Future Directions

The IPGx registry has the potential to enroll individuals who possess variants of unknown significance and novel variants. The significant clinical annotation (phenotype) that accompanies each registry record is likely to provide insights on structure–function relationships inherent in emergent variants. Additionally, over time, accumulation of a meaningful number of cases with a given novel variant has utility as a de facto cohort for future research. In fact, we expect that the IPGx registry will be a channel to recruit subjects for future research looking into the nexus of chronic disease management, pharmacogenomics, and public health; to demonstrate the value of personalized medicine approaches on public health outcomes.

The genotyping profiles and informatics in the CSN are amenable to addition of HLA insigh, a functionality that is being considered for integration into the IPGx care model and the IPGx registry in the future. This addition has great potential to inform the clinical significance of emergent HLA variants.

Oncology is a specialty from which the care of a referred population might be augmented by the IPGx model. The primary care environment utilized for the present report is not a practical context in which to develop the IPGx care platform for oncology care. A number of complementary diagnostic and drug safety paradigms for cancer therapy will be the basis for future work.

### 5.4. Opportunities

Most pharmacogenomic testing finds its way into clinical practice in a bottom-up pathway, meaning that an individual variant (genotype) or drug–gene pair is implicated in an ADR (phenotype) that is observed in the population. At that time, clinical outcomes associated with testing use cases and interventions (i.e., CYP2C19 for clopidogrel) with respect to that variant must be studied in randomized controlled trials before a recommendation for clinical testing is adopted. This approach has proven to be challenging, even for one of the most advanced areas of pharmacogenomic testing, anticoagulant therapy [40]. It takes time for the justification (pilot studies of ancillary studies piggybacked on drug registration trials) to reach a critical threshold calling for a randomized controlled trial that might ultimately demonstrate the clinical utility of testing for a given drug–gene pair. The standard innovation pathway for a pharmacogenomic use case for a drug–gene pair, can take many years for an emergent drug–gene pair to achieve reimbursement and even longer for clinical adoption [41]. The lack of a large scale clinical-genomic databases to link genotypes and drug dispensing data with outcomes is recognized as a challenge in further advancing the field of pharmacogenomics [8]. By expanding the diversity of disease and populations receiving pharmacogenomic testing beyond those fitting in narrow, existing reimbursement paradigms, the IPGx platform has the potential to produce outcome evidence for emergent drug–gene pairs and clinical use cases for pharmacogenomic testing that is supported by phenotype outcomes before (i.e., complaints in the electronic health record) and after testing (steady-state drug levels). Use of the IPGx methodology presented here would allow clinicians to make inferences from symptoms and genotyping that are in turn informed by the grounding of clinical pharmacokinetic data. This approach may provide useful insights into potential phenoconversion, a limiting challenge in relying on pharmacogenetic testing alone for clinical decision-making [42]. The clinical-genomic database of the IPGx can become a resource to inform the clinical decision making of the referring physician, and accelerate guideline maturation cycles for emergent gene–drug pairs. In the IPGx program, the informed consent and data strategy enable a simplified portrayal of bioanalytic validation of symptoms and complaints that have a pharmacologic root cause. As such, the approach is practical and actionable for a primary care provider. At the same time, the process generates a rich corpus of longitudinally integrated biological, genomic, and clinical information that is highly valuable for supporting research, practice improvement, policy, and reimbursement.

## Figures and Tables

**Table 1:** Health Data Inputs into the Clinical Semantic Network to Interrogate for Symptoms and Complaints that Might have Pharmacological Root-Cause.

Input Datum
Progress Notes (6 months)
Complaints
Active problem list
Medical History
Family History
Social History
Vitals
Vaccination History
Patient encounters (10 years)
Medication and dosing
Diagnosis codes
Billing
Quality of life questionnaires (disease specific, digital)
Continuity of care documents
Procedural notes

**Table 2:** Drug Classes, Potentially Impacted Drugs, and Genes Texted in the MolecularDx Pharmacogenomics Platform as of March 2021.

Drug Class	Potentially Impacted Drugs	Gene(s) Tested
ADHD	Atomoxetine, Amphetamines, Dexmethylphendiate, Dextroamphetamine, Lisdexamfetamine, Methylphendiate Clonidine, Guanfacine	CYP2D6, COMT
Alzheimer’s Disease	Donepezil, Galantamine Memantine	CYP2D6
Antiarrhythmics	Donepezil, Galantamine Memantine	CYP2D6
Anticancer Agents	Methotrexate, Belinostat, Erlotinib, Gefitinib, Nilotinib, Pazopanib, Azathioprine, Mercaptopurine, Thioguanine, Irinotecan, Irinotecan Liposomal	
Antidepressants, SSRIs/SNRI	Citalopram, Escitalopram, Desvenlafaxine, Duloxetine, Mirtazapine, Paroxetine, Sertraline, Venlafaxine	CYP2D6, CYP2C19
Antidepressants, Tricyclic	Amitriptyline, Clomipramine, Desipramine, Doxepin, Imipramine, Nortriptyline, Trimipramine Amoxapine, Fluoxetine, Fluvoxamine, Levomilnacipran, Maprotiline, Nefazodone, Protriptyline, Vilazodone, Vortioxetine	CYP2C9
Antidiabetics	Glimepiride, Glipizide, Glyburide, Tolbutamide, Chlorpropamide Nateglinide, Repaglinide	CYP2C9
Antiemetics	Ondansetron, Dolasetron, Metoclopramide, Palonosetron	CYP2D6
Antiepileptic	Phenytoin, Carbamazepine, Carbatrol, Eslicarbazepine, Ethosuximide, Ezogabine, Felbamate, Fosphenytoin, Gabapentin, Lacosamide, Lamotrigine, Levetiracetam, Oxcarbazepine, Perampanel, Pregabalin, Rufinamide, Tiagabine, Topiramate, Valproic Acid, Vigabatrin, Brivaracetam, Phenobarbital, Primidone, Zonisamide	CYP2C9
Antihyperlipidemic Agents	Atorvastatin, Fluvastatin, Lovastatin, Pravastatin, Pitavastatin, Simvastatin, Rosuvastatin	SLCO1B1, CYP3A4, CYP2C9
Antihypertensives	Carvedilol, Metoprolol, Irbesartan, Nebivolol, Propranolol, Timolol, Labetalol	CYP2D6, CYP2C9
Antiplatelets/Anticoagulants	Clopidogrel, Prasugrel, Ticagrelor, Warfarin, Vorapaxar, Apixaban, Dabigatran Etexilate, Edoxaban, Fondaparinux, Rivaroxaban	CYP2C19, CYP2C9, VKORC1, CYP3A5
Antipsychotics	Aripiparazole, Haloperidol, Iloperidone, Paliperidone, Perphenazine, Pimozide, Risperidone, Thioridazine, Asenapine, Brexpiprazole, Chlorpromazine, Fluphenazine, Loxapine, Lurasidone, Pimavanserin, Quetiapine, Thiothixene, Trazodone, Trifluoperazine, Ziprasidone, Clozapine, Olanzapine, TetrabenazineOther Neurological Agents: Dextromethorphan/Quinidine, Flibanserin	CYP2D6, CYP1A2
Anxiety/Insomnia	Diazepam, Clobazam, Alprazolam, Clonazepam, Lorazepam, Oxazepam	CYP2C19
Acid Related Disorders	Dexlansoprazole, Esomeprazole, Lansoprazole, Omeprazole, Pantoprazole, Rabeprazole	CYP2C19
Cardiovascular	Angiotensin II Receptor Antagonists: Azilsartan, Candesartan, Eprosartan, Irbesartan, Losartan, Olmesartan, Telmisartan, Valsartan Antianginal Agents: Ranolazine Diuretics: Torsemide	
Huntington Disease	Tetrabenazine	CYP2D6
Immunosuppressants	Tacrolimus	CYP3A5
Infections	Antifungals: Voriconazole Anti-HIV Agents: Atazanavir Antimalarials: Proguanil	
Antifugals: Voriconazole	Carisoprodol, Tizanidine, Cyclobenzaprine, Metaxalone, Methocarbamol	CYP2C19, CYP1A2
Anti-HIV Agents: Atazanavir	Methadone	CYP2B6
Antimalarials: Proguanil	Codeine, Fentanyl, Hydrocodone, Morphine, Oxycodone, Tramadol, Alfentanil, Buprenorphine, Dihydrocodeine, Hydromorphone, Levorphanol, Meperidine, Oxymorphone, Sufentanil, Tapentadol, Methadone	CYP2D6, OPRM1
Other	Bupropion, Naltrexone	COMT, OPRM1, ANKK1/DRD2
Other Analgesics	Celecoxib, Flurbiprofen, Piroxicam, Diclofenac, Ibuprofen, Indomethacin, Ketoprofen, Ketorolac, Meloxicam, Nabumetone, Naproxen, Sulindac	CYP2C9
Pain	Fibromyalgia Agents: Milnacipran	
Rheumatology	Anti-Gout Agents: AllopurinolImmunomodulators: Apremilast, Leflunomide, Tofacitinib	
Urinary Incontinence	Antispasmodics: Tolterodine, Darifenacin, Fesoterodine, Mirabegron, Oxybutynin, Solifenacin, Trospium 5-Alpha Reductase Inhibitors: Dutasteride, Finasteride Alpha Blockers: Alfuzosin, Doxazosin, Silodosin, Tamsulosin, Terazosin Phosphodiesterase Inhibitors for Erectile Dysfunction: Avanafil, Sildenafil, Tadalafil, Vardenafil	CYP2D6

**Table 3:** List of CYP2D6 alleles incorporated into the rendering of the Medication and Pharmacokinetic Pathway Summary in Figure 3.

CYP2D6 Haplotypes
* 2, * 3, * 4, * 5, * 6, * 7, * 8, * 9, * 10, * 11, * 12, * 14, * 15, * 17, * 19,
* 20, * 21, * 22, * 23, * 24, * 25, * 27, * 28, * 29, * 30, * 31, * 32, * 33,
* 34, * 35, * 36, * 37, * 38, * 39, * 40, * 41, * 42, * 43, * 44, * 45, * 46,
* 47, * 48, * 49, * 50, * 51, * 52, * 53, * 54, * 55, , * 56, * 57, * 58,
* 59, * 60, * 62, * 64, * 65, * 69, * 70, * 71, * 72, * 73, * 74, * 75, * 81,
* 82, * 83, * 84, * 85, * 86, * 87, * 88, * 89, * 90, * 91, * 94, * 95,
* 96, * 98, * 99, * 100, * 101, * 102, * 103, * 104, * 105, * 106, * 107,
* 108, * 109, * 110, * 111, * 112, * 113, * 114, * 115, * 116, * 117,
* 118, * 119, * 121, * 122, * 123, * 125, * 126, * 127, * 128, * 129,
, * 130, * 131,* 132, * 133, * 134, * 135, * 136, * 137, * 138, * 139

*: alleles.

## Data Availability

Not applicable.
